# Vaccination with live-attenuated *Toxoplasma gondii* mutants RHΔ*tkl1* and PruΔ*pp2a-c* induces protective immunity in sheep

**DOI:** 10.1186/s13071-026-07516-9

**Published:** 2026-06-16

**Authors:** Ze-Xuan Wu, Yu Kang, Wen-Bo Hao, Yu-Xuan Wang, Zhi Zheng, Shi-Bo Huang, Xiao-Nan Zheng, Xing-Quan Zhu

**Affiliations:** Shanxi Key Laboratory of Animal Disease Research, Prevention and Control, College of Veterinary Medicine, Shanxi Agricultural University, Taigu, Jinzhong, 030801 Shanxi People’s Republic of China

**Keywords:** *Toxoplasma gondii*, RHΔ*tkl1*, PruΔ*pp2a-c*, Sheep, Live attenuated vaccine, Immunization

## Abstract

**Background:**

*Toxoplasma gondii* is a globally distributed intracellular parasitic protozoan. It infects nearly all warm-blooded animals and causes a zoonotic disease of worldwide significance. Currently, the only commercially available vaccine, Toxovax^®^, is solely used for the prevention of *Toxoplasma*-induced abortion in sheep, but it has limitations such as a short shelf life and the potential of reversion to virulence. This study evaluated the safety and immune-protective efficacy of two live-attenuated strains RHΔ*tkl1* and PruΔ*pp2a-c* in sheep.

**Methods:**

Sheep were immunized via intramuscular injection in the neck with 1 × 10^7^ tachyzoites of RHΔ*tkl1* or PruΔ*pp2a-c*. Sheep were challenged orally with 5 × 10^5^ type II Pru oocysts at 28 days post-vaccination (dpv), followed by a second challenge on day 70 with 1 × 10^7^ type II Pru tachyzoites injected intramuscularly at 70 dpv. Safety and immuno-protection were evaluated by monitoring clinical symptoms and body temperatures, *T. gondii*-specific IgG antibody levels, histopathological changes, immunohistochemistry, brain cysts, parasite load, and mouse bioassay results.

**Results:**

The results demonstrated that both knockout strains induced only transient fever. Following immunization and subsequent challenge with Pru oocysts, the *T. gondii*-specific IgG antibody levels in sheep increased rapidly and remained elevated for an extended period. Histopathological analysis indicated mild organ lesions in heart, liver and lung tissues among immunized infected sheep, whereas non-immunized infected sheep exhibited severe widespread inflammation. Immunohistochemical analysis of brain tissue revealed significantly lower values for four parameters (positive cell ratio, density, histochemistry score, immunoreactive score) in immunized groups (*P* < 0.01). A significant reduction in brain cysts was observed in immunized and challenged sheep (*P* < 0.01) compared with unimmunized and challenged sheep. The parasite burden of *T. gondii* in heart tissue was significantly reduced (*P* < 0.01). Compared with mice inoculated with sheep brain tissue from unimmunized groups challenged with *T. gondii* Pru oocysts and tachyzoites, mouse bioassay results showed that the mice inoculated with sheep brain tissue from groups immunized with RHΔ*tkl1* or PruΔ*pp2a-c* tachyzoites and subsequently challenged with *T. gondii* Pru oocysts and tachyzoites exhibited a significantly lower proportion of positive genomic *T. gondii* DNA in the brain (*P* < 0.001), as well as significantly reduced levels of *T. gondii*-specific antibody IgG in the serum (*P* < 0.0001). Similarly, mice inoculated with sheep visceral tissue from the same immunized and challenged groups also showed a significantly reduced proportion of positive genomic *T. gondii* DNA in the brain (*P* < 0.0001) and significantly reduced levels of *T. gondii*-specific antibody IgG in the serum (*P* < 0.0001).

**Conclusions:**

The gene knockout strains RHΔ*tkl1* and PruΔ*pp2a-c* showed a certain degree of safety in sheep, and they induced strong humoral and cellular immune responses in sheep, significantly mitigating acute infection symptoms and tissue damage. Notably, PruΔ*pp2a-c* showed greater potential in suppressing cyst formation. Both strains are potential attenuated candidates against sheep toxoplasmosis.

**Graphical Abstract:**

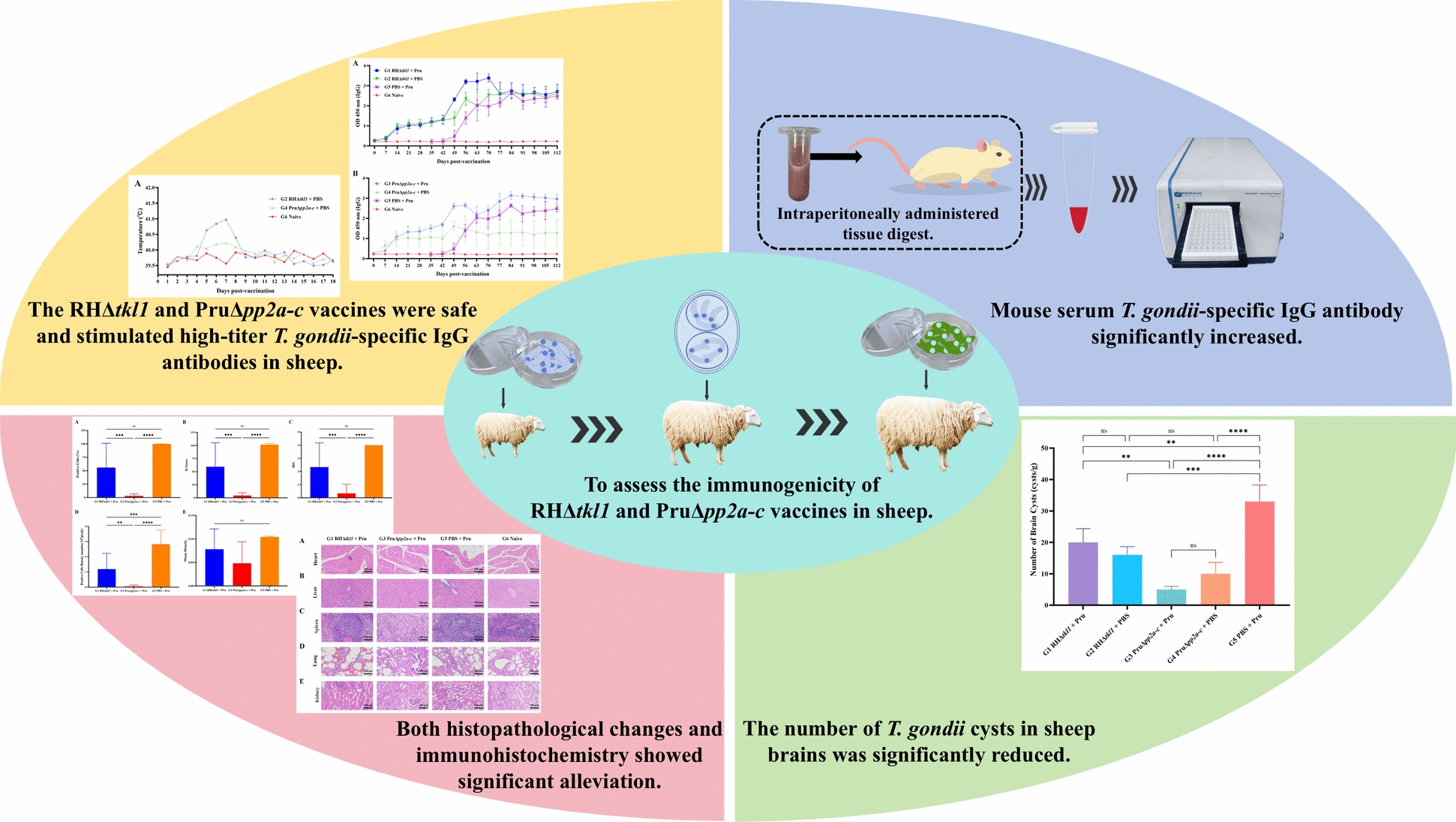

**Supplementary Information:**

The online version contains supplementary material available at 10.1186/s13071-026-07516-9.

## Background

*Toxoplasma gondii* is a member of the Apicomplexa, which includes zoonotic protozoans such as *Plasmodium* species and *Cryptosporidium* spp. [1–3]. *T. gondii* is an obligate intracellular parasitic protozoan, a unicellular eukaryote that requires a host cell for survival [4, 5]. *T. gondii* is considered a very successful parasite, partly because it can infect almost all warm-blooded animals [6]. During the life cycle of *T. gondii*, the three stages capable of infecting cells are the tachyzoite, bradyzoite, and sporozoite [7]. Approximately one-third of global population has been infected with *T. gondii*, with most cases being asymptomatic; and serologic investigations showed that wild carnivores such as bears, felids, fox, raccoons and skunks are commonly infected with *T. gondii* [8]. However, *Toxoplasma* poses severe risks to pregnant individuals, pregnant livestock, and immunocompromised hosts, potentially leading to miscarriage, stillbirth, neonatal deformities (e.g., hydrocephalus, visual impairment, intellectual disability), and complications such as encephalitis, retinochoroiditis, and pneumonia, with severe cases resulting in death [9–13].

The genus *Toxoplasma* contains only one species, *T. gondii*, that can be grouped into various genotypes (such as types Ⅰ, Ⅱ, Ⅲ and 12) [14]. Type 12 was recently isolated from wild animals [15, 16]. In North America and Europe, clonal Type III and II strains are predominant [17]. Humans are mainly infected with Type II *T. gondii* and it is widely distributed in Europe, North America, and Africa [18]. Chinese 1 (ToxoDB#9) genotype accounted for 78% of the total *T. gondii* strains isolated from animals and humans in China [19–21]. *T. gondii* exhibits a broad host range, encompassing mammals, birds, humans and amphibians in the rare case [22]. Susceptibility to *T. gondii* infection and the resulting clinical manifestations vary significantly among different animal species [23]. Among domestic animals, toxoplasmosis poses the most severe threat to pigs and sheep [24–27]. Infected animals may present with a range of clinical signs such as high fever, dyspnea, and locomotor disorders. In pregnant dams, infection can lead to abortion and stillbirth [9]. As livestock and poultry serve as major sources of meat for human consumption, chronic or subclinical infected animals can transmit the parasite through the food chain, constituting a significant source of human infection with *T. gondii* [28, 29].

Both sheep and goats are permissive hosts for *T. gondii* [30–32]. Infection in these animals can manifest with a range of clinical signs, with its primary impact being the induction of reproductive disorders. These include early embryonic death and resorption, fetal mummification, abortion, stillbirth and neonatal mortality, causing substantial economic losses to the sheep and goat industries [27, 33]. Furthermore, *T. gondii* present in ovine and caprine tissues—such as meat, offal, blood, secretions, and milk—can serve as a significant source of infection for other animals and humans [34]. Currently, the development of *T. gondii* vaccines has emerged as an effective strategy for infection prevention and control [35]. The commercially available Toxovax^®^, based on the live-attenuated S48 strain, is the only licensed veterinary vaccine for preventing ovine abortion and fetal infection caused by *T. gondii*. This vaccine strain was originally isolated from an aborted lamb in New Zealand and subsequently attenuated through extensive serial passage in mice [36, 37]. However, the commercial application of this attenuated strain is constrained by limitations such as a suboptimal stability profile and the potential for regained virulence [27].

Live attenuated parasite strains generated by gene knockout are widely regarded as a promising strategy for preventing toxoplasmosis in livestock [38]. Previous studies assessed the effectiveness of a mutant strain of *T. gondii* (RH strain) lacking the mic1 and mic3 genes (Mic1-3KO) against *Toxoplasma* abortion in sheep, found that Mic1-3KO was as effective as S48 (the strain used as a live vaccine for sheep, Toxovax®), and demonstrated that Mic1-3KO is a potent vaccine candidate [39, 40]. The TKL1 gene encodes a tyrosine kinase-like protein that regulates *Toxoplasma*’s lytic cycle [41]. It is an essential virulence factor with a potential immunogenicity [41]. PP2A-C is a catalytic subunit of the *Toxoplasma* protein phosphatase 2A holoenzyme which regulates starch metabolism and bradyzoite differentiation in *T. gondii* via dephosphorylation [42]. Its deletion prevents the transition of tachyzoites to bradyzoites and attenuated the virulence of *T. gondii* in mice [42].

Two live-attenuated *T. gondii* mutants, RHΔ*tkl1* and PruΔ*pp2a-c*, have been shown to provide strong protective immunity against *T. gondii* infection in murine models [41, 43]. Nevertheless, whether they can induce a similar protective immune response in sheep remains to be determined. Consequently, this study was designed to evaluate the protective efficacy conferred by immunization with these attenuated knockout strains against sheep toxoplasmosis, while also exploring their viability as potential vaccine candidates for sheep toxoplasmosis.

## Methods

### Animals and parasites

Twenty-four lambs aged approximately four months were purchased from Shuomeiyang Sheep Industry Co., Ltd., Jinzhong City, Shanxi Province. These lambs were tested negative for *T. gondii* IgG by ELISA. They were randomly assigned to individual pens (four animals each) and left to acclimatize for 7 days before the experiment. During the duration of the experimental period, a standard commercial sheep feed was provided to all animals, and water was available ad libitum. Eight-week-old female Porton mice (a minimally inbred strain) were purchased from Beijing Sibeifu Biotechnology Co. Ltd. [44]. Mice were kept under specific pathogen-free conditions with 50–60% humidity and 22 °C, and fed with food and water ad libitum. All mice were acclimatized for one week before the beginning of the experiments. Animal experiments were performed strictly according to the principles of minimizing animal suffering and protecting animal welfare as much as possible.

The tachyzoites of *T. gondii* Type II Pru strain, the constructed gene knockout RHΔ*tkl1* and PruΔ*pp2a-c* strains were replicated in human foreskin fibroblast (HFF) cells, maintained in DMEM containing 2% fetal bovine serum (FBS), and cultured in an incubator at 37 °C with 5% CO_2_ [41, 42]. The infectious oocysts of *T. gondii* Pru strain were kept suspended in 2% H_2_SO_4_ at 4 ℃ in the Laboratory of Parasitic Diseases, College of Veterinary Medicine, Shanxi Agricultural University (Additional file 1: Fig. S1.) [43]. The maintenance and care of experimental animals, including sheep and mice, complied with the requirements of the Institutional Animal Care and Use Committee of Shanxi Agricultural University (Approval No.: SXAU-EAW-2021XM121001).

### Sheep vaccination and challenge

**Fig. 1:**
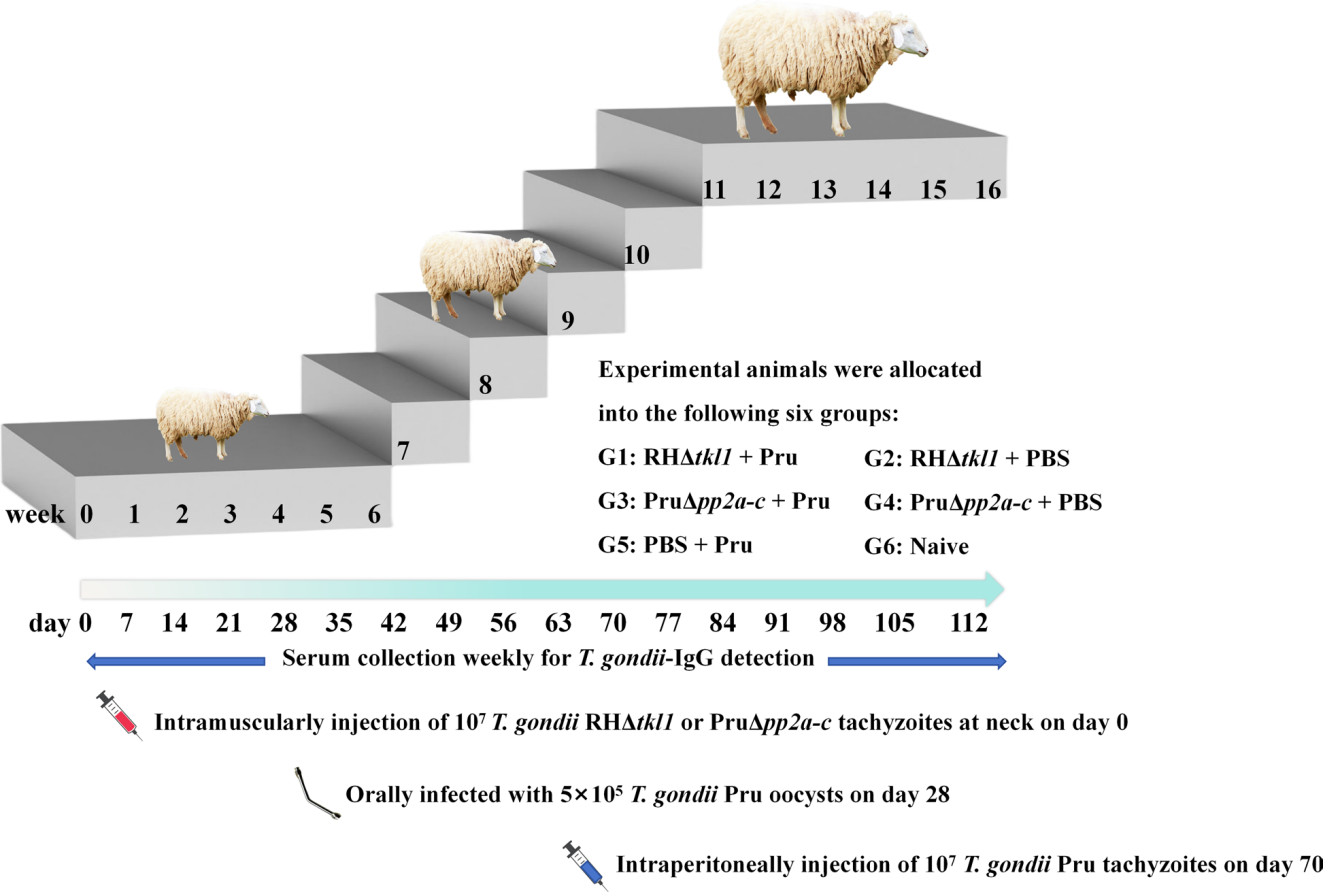
Scheme of the vaccination and challenge experiment. The experimental timeline (days 0–112) is presented

The 24 sheep were divided into six groups (G) depending on experimental immunization and challenge infection (see Table 1 and Fig. 1). On day 0, sheep in G1 and G2 were injected intramuscularly in the neck with 10⁷ tachyzoites of *T. gondii* RHΔ*tkl1*, while sheep in G3 and G4 were similarly injected intramuscularly in the neck with 10^7^ tachyzoites of *T. gondii* PruΔ*pp2a-c*. 28 days post-vaccination (dpv), animals in G1, G3 and G5 were orally challenged with 5 × 10^5^ T*. gondii* oocysts of the Pru strain. On 70 dpv, animals in groups G1, G3 and G5 were challenged intraperitoneally with 1 × 10^7^ tachyzoites of the *T. gondii* Pru strain. Following immunization with RHΔ*tkl1* or PruΔ*pp2a-c* and subsequent challenge with Pru oocysts and Pru tachyzoites, clinical signs in sheep were monitored and rectal temperatures were recorded daily. The timeline of the experimental design is shown in Fig. 1.

**Table 1:** Experimental design: animal grouping, vaccination with attenuated *Toxoplasma gondii* strains, and challenge infection

Group	Vaccination and/or challenge		
	Day 0 (vaccination)	Day 28 (primary challenge)	Day 70 (secondary challenge)
1 (*n* = 4)	1 × 10^7^ RHΔ*tkl1* tachyzoites	5 × 10^5^ Pru oocysts (in 5 mL PBS)	1 × 10^7^ Pru tachyzoites (in 1 mL PBS)
2 (*n* = 4)	1 × 10^7^ RHΔ*tkl1* tachyzoites	5 mL PBS	1 mL PBS
3 (*n* = 4)	1 × 10^7^ PruΔ*pp2a-c* tachyzoites	5 × 10^5^ Pru oocysts (in 5 mL PBS)	1 × 10^7^ Pru tachyzoites (in 1 mL PBS)
4 (*n* = 4)	1 × 10^7^ PruΔ*pp2a-c* tachyzoites	5 mL PBS	1 mL PBS
5 (*n* = 4)	1 mL PBS	5 × 10^5^ Pru oocysts (in 5 mL PBS)	1 × 10^7^ Pru tachyzoites (in 1 mL PBS)
6 (*n* = 4)	1 mL PBS	5 mL PBS	1 mL PBS

### Collection of tissue samples

Blood sampling was carried out weekly during the experiment, stored at 4 ℃ for 2 h, and then centrifuged at 3000 × *g* for 10 min. The separated serum was stored at – 20 ℃ before further analysis. On 112 dpv, sheep were euthanized by intravenous administration of a barbiturate. Samples from skeletal muscle (including the longissimus dorsi and psoas major) and from the brain, spleen, heart, lung, liver, kidney, and lymph nodes (right prescapular and distal jejunal) were collected. Tissue samples measuring 2 × 2 cm, were collected, fixed in 10% buffered formalin solution for 5 days, and subsequently processed using standard histopathological methods for hematoxylin and eosin (HE) staining and immunohistochemistry (IHC) [36]. The remaining fresh tissue samples were stored at − 20 °C for subsequent use.

### Detection of sheep *T. gondii* IgG by enzyme linked immunosorbent assay (ELISA)

Following the manufacturer’s instructions, sheep serum samples were analyzed for anti-*T. gondii* IgG antibodies using the ID Screen Toxoplasmosis Indirect Multi-species ELISA kit (ID.vet, Montpellier, France) [44]. The ELISA was considered valid only when the mean optical density (OD) of the positive controls exceeded 0.35, and the ratio of the mean OD for positive to negative controls was greater than 3. Results were interpreted based on the percent seropositivity (SP), with an SP value ≥50% defined as a positive result.

### Tissue HE and IHC staining

Tissue samples (including heart, liver, spleen, lung, kidney, brain, psoas major muscle, longissimus dorsi muscle, superficial cervical lymph nodes and jejunal lymph nodes) collected from sheep were fixed in 10% neutral buffered formalin (NBF) at room temperature for 24 h. After fixation, tissue samples were dehydrated through a graded series of ethanol (70, 80, 90, and 100%), with each step lasting 30 min. Subsequently, they were cleared in xylene (two changes, 30 min each) and infiltrated with molten paraffin in a 58–60 °C oven for 1–2 h. The embedded tissues were then sectioned at 4–6 µm using a microtome. Finally, the sections were stained with HE for microscopic evaluation of histopathological alterations [45].

The brain tissue sections used for HE staining were further processed for IHC to detect *T. gondii* antigen, using a specific polyclonal antibody against both tachyzoites and bradyzoites [46]. Tissue sections were deparaffinized, rehydrated, and subjected to heat-induced epitope retrieval in citrate buffer. After quenching endogenous peroxidase activity and blocking with BSA, sections were incubated with primary antibody overnight at 4 °C, followed by HRP-conjugated secondary antibody. Detection was performed with DAB chromogen, counterstained with hematoxylin, dehydrated, cleared, and mounted for microscopic examination and analysis. The IHC procedure was carried out using Dako EnVision^®^ + System-HRP (Dako North America Inc., Carpinteria, USA) [47]. Expression and distribution of *T. gondii* in tissues were quantified on stained sections using immunohistochemical scoring methods, which included positive cell ratio, positive cell density, histochemistry score (H-Score), mean optical density, and immunoreactive score (IRS) [48–50].

### The microscopic examination and enumeration of *T. gondii* cysts in sheep brain tissue

To assess the cyst burden, sheep were euthanized at 112 dpv. Cysts were quantified as previously described [51]. Briefly, the brain tissue from sheep in G1, G2, G3, G4, G5 and G6 groups was weighed, and 5 g of uniformly sampled brain tissue was placed in a mortar and homogenized to a paste-like consistency. A volume of 5 mL PBS was added to resuspend the homogenate. The brain homogenate was first transferred to a 15 mL centrifuge tube, followed by the careful addition of an equal volume of Ficoll-Paque^™^ PREMIUM (1.084) (Merck KGaA, GE17-5446-02, Germany) density gradient medium along the tube wall. Centrifugation was performed at 1750 × *g* for 30 min. The bottom layer, which contained the *T. gondii* cysts, was gently aspirated. Microscopic examination and enumeration were conducted to calculate the cyst burden in each sheep brain sample.

### Detection of *T. gondii* B1 gene by reverse transcription quantitative polymerase chain reaction (RT-qPCR)

The genomic DNA of tissues (including heart, liver, spleen, kidney, brain, psoas major muscle and longissimus dorsi muscle) and *T. gondii* tachyzoites were extracted using the TIANamp Genomic DNA Kit (Tiangen Biotech, DP304-03, Beijing, China). *T. gondii* DNA was quantified in these DNA samples by amplifying the highly conserved B1 gene via RT-qPCR [52]. RT-qPCR was performed using forward primer (F5′-AGGGACAGAAGTCGAAGGGG-3′) and reverse primer (R5′-GCAGCCAAGCCGGAAACATC-3′) [52], amplifying a 164-bp fragment of B1 gene with SYBR Premix Ex *Taq* (Takara, DRR041A, Dalian, China). The following amplification protocol was applied: 10 min at 95 °C, 40 cycles at 95 °C for 15 s (denaturation), 62 °C for 45 s (annealing), and 72 °C for 30 s (amplification). The normalized sample DNA was then subjected to quantitative PCR (qPCR) using SYBR Premix Ex *Taq* to detect the *T. gondii* B1 gene and determine its cycle threshold (CT) values. Subsequently, parasite load was determined using the standard curve analysis based on the Lg (tachyzoite number) and the corresponding CT values of different gradient tachyzoites.

### Mouse bioassay

Tissues from sheep in G1, G3 and G5 for mouse bioassay were grouped differently. Based on the tissue type, collected tissues were divided into the following groups: “Brain” (50 g of brain), and “Viscera” (a 50 g pooled sample which included 10 g each of heart, liver, spleen, lung and kidney) [44]. These tissues were digested with acid/pepsin employing a method reported previously [53]. After the tissue homogenate was centrifuged for 10 min at 1200 *g*, the supernatant was poured off gently and the final pellet was resuspended in 3 mL sterile saline (which contained 400 µg/mL penicillin and 400 units/mL streptomycin). Three mice were intraperitoneally injected with 400 µL of each inoculate. Throughout the six-week course of infection, clinical symptoms and mortality rates in mice were observed and recorded.

Mice surviving to the end of the six-week bioassay were euthanized. Brain was stored separately in a sterile vial containing 1 mL PBS for subsequent DNA extraction and PCR analysis. Detection of *T. gondii* in mouse brain tissue were performed by PCR targeting the *T. gondii* B1 gene, according to a previously described protocol [42]. To assess humoral immune responses, *T. gondii*-specific antibody in mouse serum were detected via ELISA as previously described [54, 55]. Briefly, wells of a 96-well plate were coated with 100 μl of STAg (1 μg/well) by incubation at 37 °C for 2 h, followed by an overnight coating at 4 °C. After coating, the plate was washed three times with 0.5% phosphate-buffered saline with Tween 20 (PBST) and patted dry. Non-specific binding sites were then blocked with 5% bovine serum albumin (BSA) at 37 °C for 1 h, followed by another washing step. Serum samples diluted 1:100 in 1% BSA were added and incubated at 37 °C. Subsequently, wells were incubated with HRP-conjugated goat anti-mouse IgG (Abcam, AB97040, Cambridge, UK) diluted 1:3000. After three washes, color development was initiated by adding TMB (3, 3′, 5, 5′-Tetramethylbenzidine) Chromogen Solution (Beyotime Biotech, P0209-100 ml, Shanghai, China). The reaction was terminated with 2% sulfuric acid upon color stabilization, and the optical density was measured at 450 nm.

### Statistical analysis

Experimental data were acquired for three biological replicates and analyzed using GraphPad Prism 8.0 (GraphPad Software Inc., La Jolla, CA, USA). A two-tailed, unpaired Student’s *t*-test was used to examine the significance of differences between two groups, including antibody levels and parasite burden. Quantitative variables were presented as the means ± standard deviations (SD). *P* < 0.05 was considered statistically significant, and *P* < 0.01, < 0.001, < 0.0001 represented different degrees of significance.

## Results

### Clinical observations in sheep

**Fig. 2:**
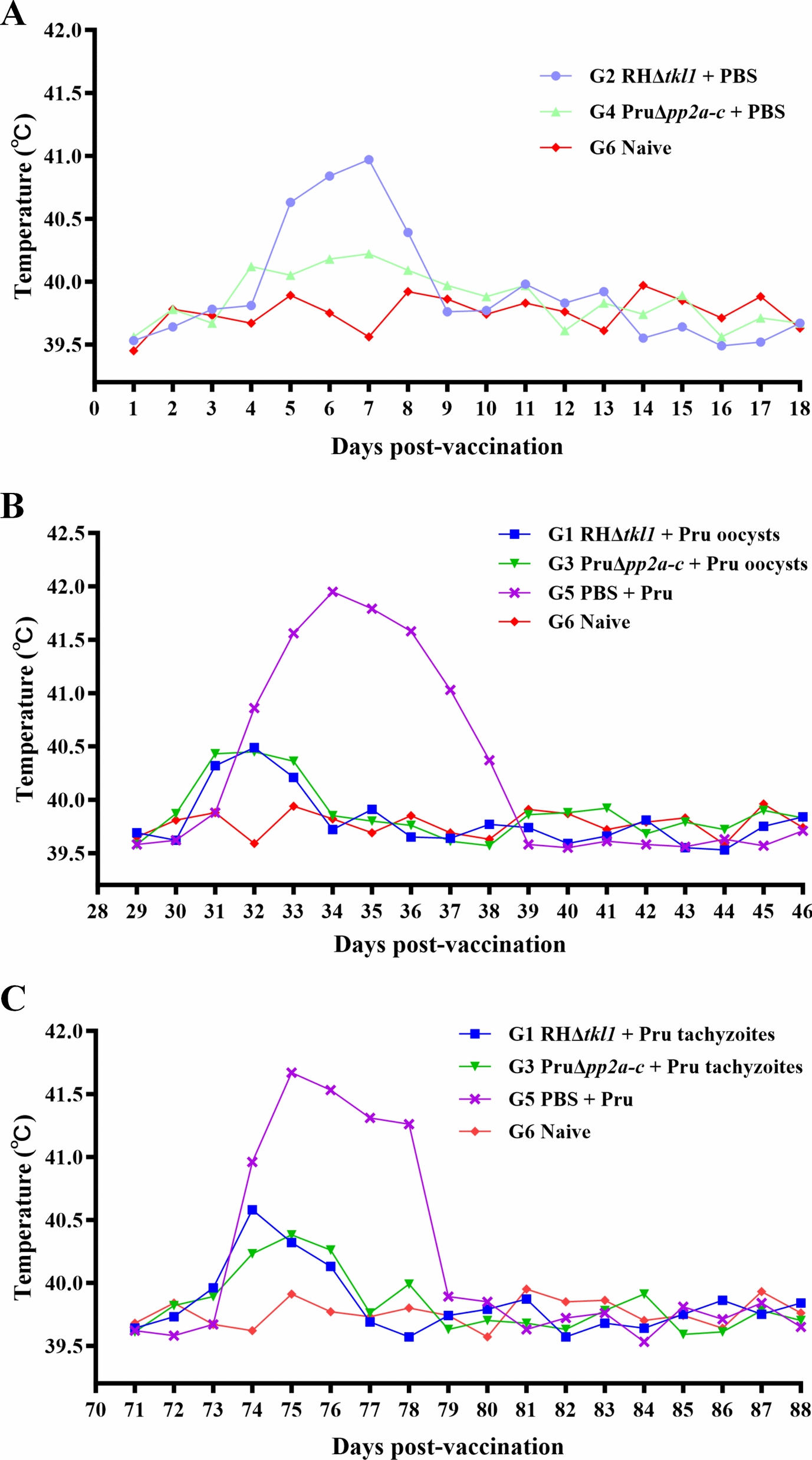
Mean body temperature of sheep in each experimental group post-vaccination and post-challenge. **A** Temperature after vaccination with RHΔ*tkl1* or PruΔ*pp2a-c* tachyzoites. **B** Temperature after challenge with oocysts of *Toxoplasma gondii* Pru strain. **C** Temperature after second challenge with tachyzoites of* T. gondii* Pru strain

After immunization with RHΔ*tkl1* or PruΔ*pp2a-c* tachyzoites, body temperatures of sheep in groups G2, G4, and G6 were monitored. After primary challenge with Pru oocysts and secondary challenge with Pru tachyzoites, body temperatures of sheep in groups G1, G3, G5, and G6 were monitored. Following immunization with RHΔ*tkl1* tachyzoites, sheep in group G2 showed an increase in body temperature that peaked at 7 days post-vaccination (dpv), then declined and returned to normal by 9 dpv (Fig. 2A). In the group G4 immunized with PruΔ*pp2a-c* tachyzoites, body temperature began to rise at 3 dpv and also returned to normal by 9 dpv (Fig. 2A). In the control group G6, the mean rectal temperature remained constant within physiological levels throughout the monitoring period (Fig. 2A–C).

After challenge with Pru oocysts at 28 dpv, groups G1 and G3 exhibited only a mild and transient temperature elevation lasting 4 days (Fig. 2B). In contrast, the group G5 showed a continuous temperature increase that peaked at 41.9 ℃ by 34 dpv, followed by a progressive decline to the normal baseline by 39 dpv (Fig. 2B).

Following the second challenge with Pru strain tachyzoites at 70 dpv, groups G1 and G3 exhibited only a mild and transient fever lasting 5 days (Fig. 2C). However, the G5 group showed a continuous temperature increase, peaking at 41.67 °C by 75 dpv, then progressively declining to the normal baseline by 80 dpv, accompanied by symptoms of depression and increased respiratory rate (Fig. 2C).

### Vaccination with the RHΔ*tkl1* and PruΔ*pp2a-c* strains triggered a strong humoral response in sheep

**Fig. 3:**
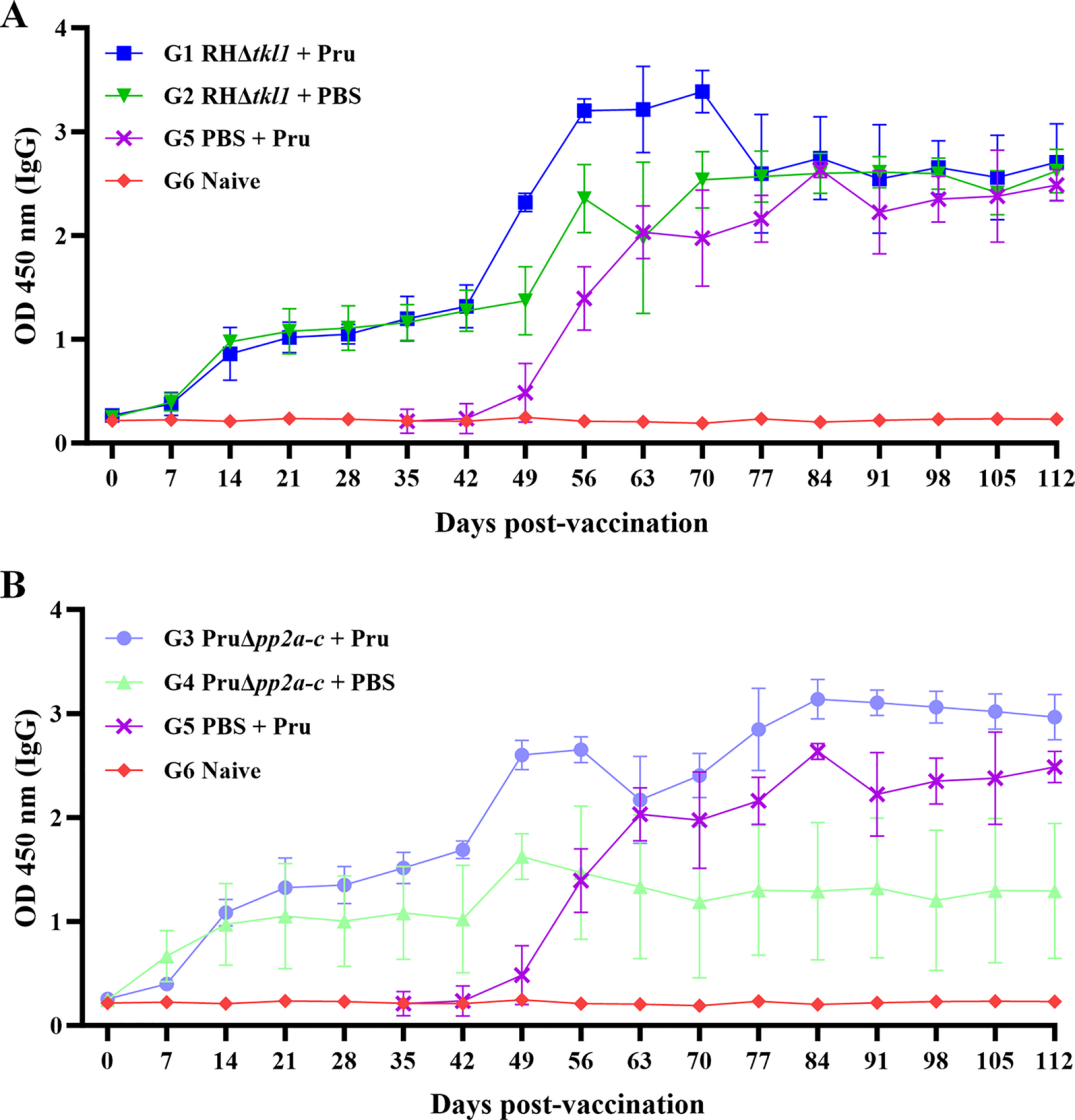
*T. gondii*-specific IgG levels in sheep serum post-vaccination and post-challenge. Serum IgG levels were measured by ELISA from day 0 to day 112 after immunization. **A** Groups vaccinated with RHΔ*tkl1* tachyzoites along with naive and non-vaccinated groups. **B** Groups vaccinated with PruΔ*pp2a-c* tachyzoites along with naive and non-vaccinated groups

Vaccination with RHΔ*tkl1* or PruΔ*pp2a-c* induced a strong humoral response in sheep. Following vaccination with RHΔ*tkl1*, *T. gondii*-specific antibodies in groups G1 and G2 increased gradually (Fig. 3A). After the first challenge with Pru strain oocysts at 28 dpv, antibody levels in group G1 rose rapidly, peaking around 70 dpv (Fig. 3A). Following the second challenge with Pru strain tachyzoites at 70 dpv, the antibody titer in group G1 declined and became almost equal to that in group G2, and both groups maintained elevated level until the end of the study (Fig. 3A). Compared to group G5, vaccination in group G1 induced an effective anamnestic response, resulting in significantly higher and sustained antibody levels, indicative of a protective immunological memory (Fig. 3A). The control group G6 maintained baseline or negligible levels of specific IgG throughout the study, confirming the absence of specific immune stimulation or environmental exposure (Fig. 3A).

The overall trend of *T. gondii*-specific antibodies following immunization with PruΔ*pp2a-c* was consistent with that observed after RHΔ*tkl1* vaccination (Fig. 3B). After challenge with Pru strain oocysts at 28 dpv, antibody levels in group G3 immunized with PruΔ*pp2a-c* increased rapidly, reaching their peak earlier, by 56 dpv (Fig. 3B). Following the second challenge with Pru strain tachyzoites at 70 dpv, the *T. gondii*-specific antibodies in group G3 showed a slight and transient increase, and then remained at that level until the end of the study (Fig. 3B). After the second challenge with Pru strain tachyzoites, the overall trend of *T. gondii*-specific IgG antibody levels in group G3 was significantly higher than that in group G1 (*P* < 0.05) (Fig. 3).

### Histopathology and IHC

In heart tissue of the G5 group, myocardial necrosis was observed in the myocardial layer, characterized by nuclear pyknosis and cytoplasmic disintegration. The G1 and G3 groups exhibited moderate myocardial atrophy in the myocardial layer, along with scant lymphocytic infiltration around interstitial blood vessels (Additional file 2: Fig. S2A). In liver tissue of the G5 group, hepatocellular edema and fatty degeneration were present, featuring pale and loose cytoplasm, cellular swelling, and mild dilation of numerous hepatic sinusoids. The G1 and G3 groups showed fatty degeneration of hepatocytes, with small cytoplasmic vacuoles visible (Additional file 2: Fig. S2B). Spleen tissue in the G5 group displayed dilation of spleen sinusoids and scattered infiltration of granulocytes in the marginal zone. In the G1 and G3 groups, the splenic capsule appeared unremarkable, with mild dilation of a few sinusoids observed (Additional file 2: Fig. S2C). In the lungs of the G5 group, prominent lymphocytic infiltration was seen around bronchioles and some blood vessels, along with scant granulocytic infiltration in the alveolar walls and mild thickening of alveolar septa. The G1 and G3 groups exhibited smooth pleural surfaces without significant abnormalities, with marked lymphocytic infiltration around multiple bronchioles and a few blood vessels, and irregular arrangement of epithelial cells in some bronchioles (Additional file 2: Fig. S2D). Kidney tissue in the G5 group showed hydropic degeneration of tubular epithelial cells, with pale and loose cytoplasm, mild dilation of many renal tubules, and occasional tubular atrophy with reduced volume. The G1 and G3 groups displayed scant hydropic degeneration of tubular epithelial cells, featuring pale and loose cytoplasm, and mild dilation of a few tubules (Additional file 2: Fig. S2E).

Brain tissue in the G5 group exhibited numerous shrunken and hyperchromatic neurons with reduced and deformed morphology, indistinct nuclear and cytoplasmic boundaries, and frequent perivascular space enlargement. The G1 and G3 groups occasionally showed several shrunken and hyperchromatic neurons with cellular shrinkage and deformation (Additional file 3: Fig. S3A). In the psoas muscle of the G5 group, scant myocytes exhibited hydropic degeneration. The G1 and G3 groups occasionally displayed scattered lymphocytic infiltration around interstitial blood vessels (Additional file 3: Fig. S3B). The longissimus dorsi muscle in the G5 group showed multiple small focal lymphocytic infiltrates in the interstitium. The G1 and G3 groups exhibited scant hydropic degeneration of myocytes, with pale and loose cytoplasm (Additional file 3: Fig. S3C). In the superficial cervical lymph nodes of the G5 group, extensive smooth muscle hyperplasia, thickening, and scattered distribution throughout the medulla were observed. The G1 and G3 groups showed indistinct boundaries of lymphoid follicles and rare germinal centers (Additional file 3. Fig. S3D). Jejunal lymph nodes in the G5 group occasionally exhibited germinal centers and eosinophilic material, with mild dilation of medullary sinuses in small areas and loosely arranged cells. The G1 and G3 groups displayed extensive dilation of medullary sinuses and scant smooth muscle scattered within the medulla (Additional file 3. Fig. S3E).

**Fig. 4:**
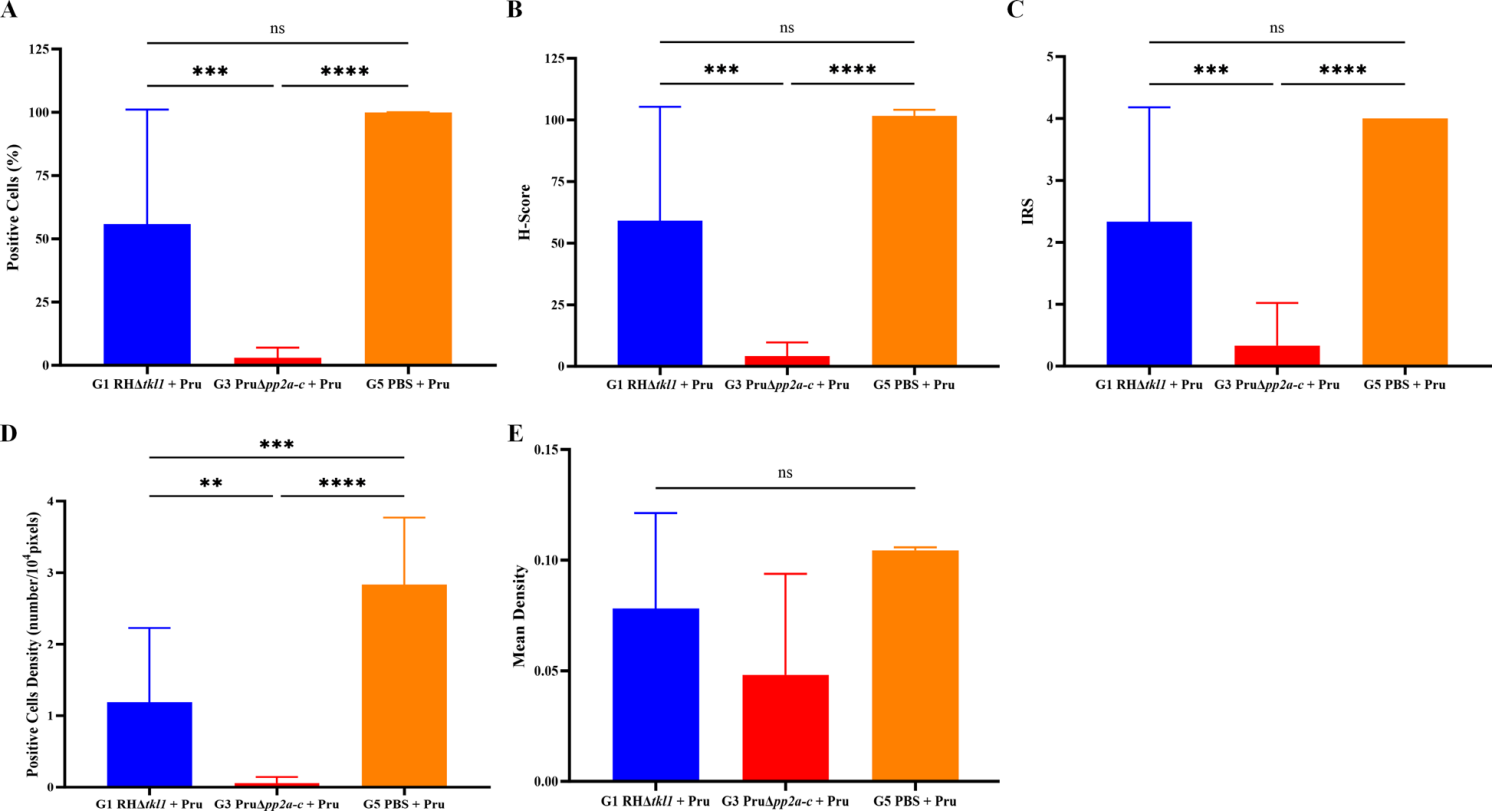
Immunohistochemical (IHC) analysis of brain tissue from challenged sheep. Analysis of the significance of five relevant parameters, positive cell ratio (**A)**, positive cell density (**B)**, histochemistry score (**C)**, mean optical density (**D)**, and immunoreactive score (**E)** in immunohistochemistry. ****P < 0.0001, ***P < 0.001, **P < 0.01, *P < 0.05

IHC staining was performed on sheep brain tissues from groups G1, G3, and G5, with five relevant parameters were analyzed (Additional file 4: Fig. S4). The positive cell ratio, H-Score, and IRS were all significantly higher in groups G1 and G5 than in group G3 (*P* < 0.001) (Fig. 4A–C). Similarly, positive cell density was significantly elevated in groups G1 and G5 compared with group G3 (*P* < 0.01) (Fig. 4D). In contrast, the mean optical density did not differ significantly among the three groups (*P* > 0.05) (Fig. 4E).

### Significant reduction in brain cyst formation following immunization with RHΔ*tkl1* or PruΔ*pp2a-c*

**Fig. 5:**
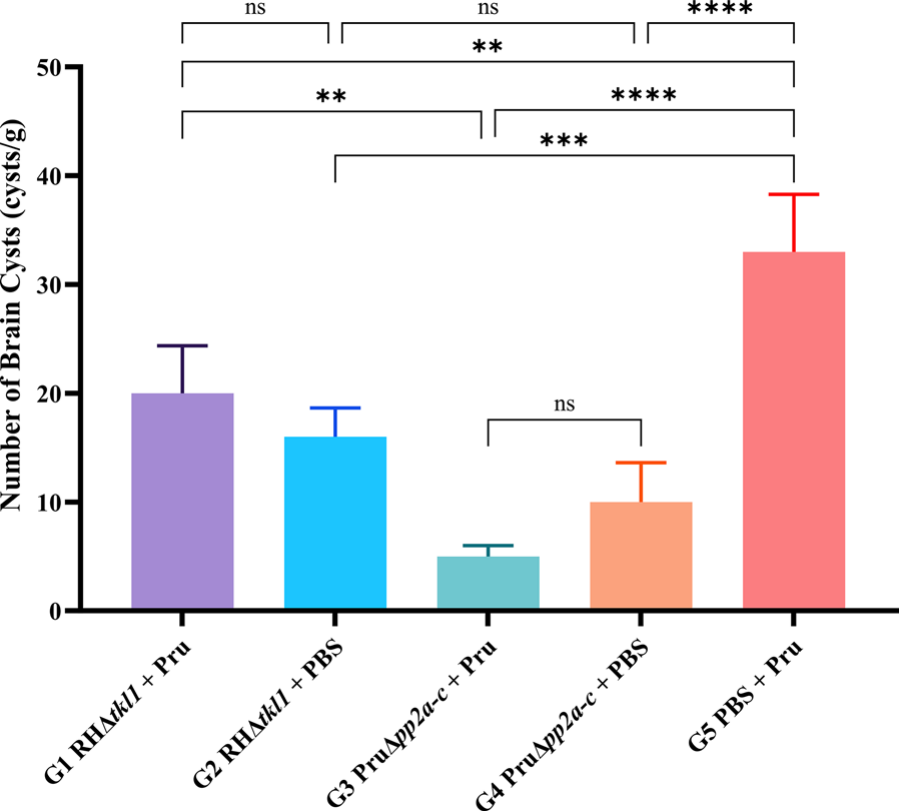
Quantitative analysis of mean brain cyst counts. ****P < 0.0001, ***P < 0.001, **P < 0.01, *P < 0.05

*T. gondii* tissue cysts were microscopically detected in brain tissues from groups G1, G2, G3, G4, G5 and G6, whereas no cysts were observed in the brains of animals from the blank control group G6 (Additional file 5: Fig. S5). Quantitative analysis revealed the following mean brain cyst counts (per g): G1 (20 cysts/g), G2 (16 cysts/g), G3 (10 cysts/g), G4 (5 cysts/g), G5 (33 cysts/g) and G6 (0 cysts/g). Compared to the high-burden group G5, the mean brain cyst counts were significantly lower in groups G1, G2, G3, and G4 (*P* < 0.01) (Fig. 5). Furthermore, group G3 showed a significantly lower cyst burden than group G1 (*P* < 0.01) (Fig. 5). No statistically significant differences were observed between groups G1 and G2 (*P* > 0.05), or between groups G3 and G4 (*P* > 0.05) (Fig. 5).

### Effective reduction of tissue *T. gondii* load in sheep immunized with RHΔ*tkl1* or PruΔ*pp2a-c*

**Fig. 6:**
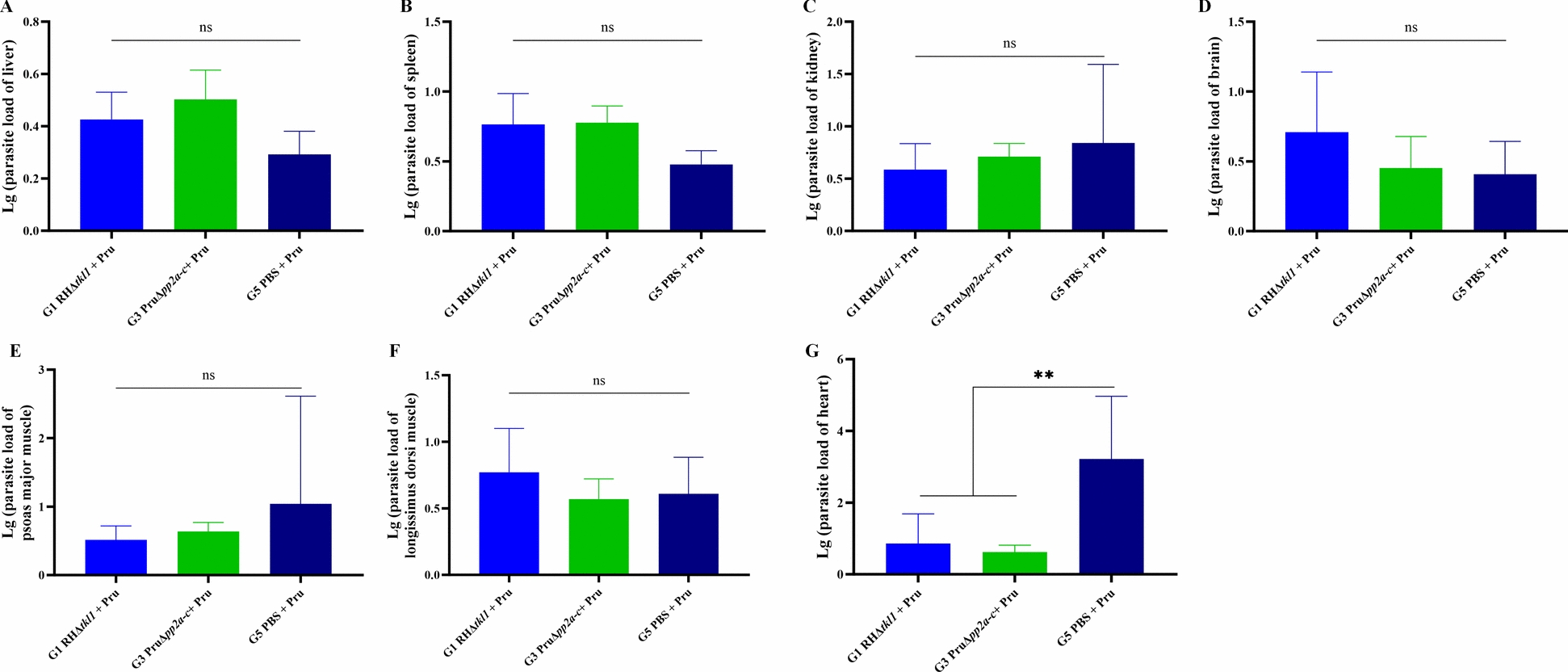
Parasite burden in tissues of vaccinated sheep. Parasite load was quantified by reverse transcription quantitative polymerase chain reaction (RT-qPCR) in the liver (**A)**, spleen (**B)**, kidney (**C)**, brain **D**, psoas major muscle (**E)**, longissimus dorsi muscle (**F)** and heart (**G)** of sheep immunized with 1 × 10^7^ RHΔ*tkl1* or PruΔ*pp2a-c* tachyzoites at day 112 post-vaccination with 1 × 10^7^ tachyzoites of RHΔ*tkl1* or PruΔ*pp2a-c*. ****P < 0.0001, ***P < 0.001, **P < 0.01, *P < 0.05

*T. gondii* load showed no significant differences among groups G1, G3, and G5 in the liver, spleen, kidney, brain, psoas major muscle, and longissimus dorsi muscle tissues of sheep (*P* > 0.05) (Fig. 6A–F). In heart tissue, however, the *T. gondii* load was significantly lower in groups G1 and G3 compared to group G5 (*P* < 0.01) (Fig. 6G).

### Detection of *T. gondii* DNA from mouse brains following bioassay

All mice survived until the end of the sixth week post-inoculation. The *T. gondii* positivity rates in mice inoculated with brain tissue were 78.8% (7/9) for group G5, 66.7% (6/9) for group G1, and 44.4% (4/9) for group G3. Compared with the group G5, the positive rates in the G1 and G3 groups were significantly lower (*P* < 0.001). For mice inoculated with visceral tissues, the positivity rates were 67.7% (6/9) in group G5, 33.3% (3/9) in group G1, and 22.2% (2/9) in group G3. Compared with the group G5, the positive rates in the G1 and G3 groups were significantly lower (*P* < 0.0001).

**Fig. 7:**
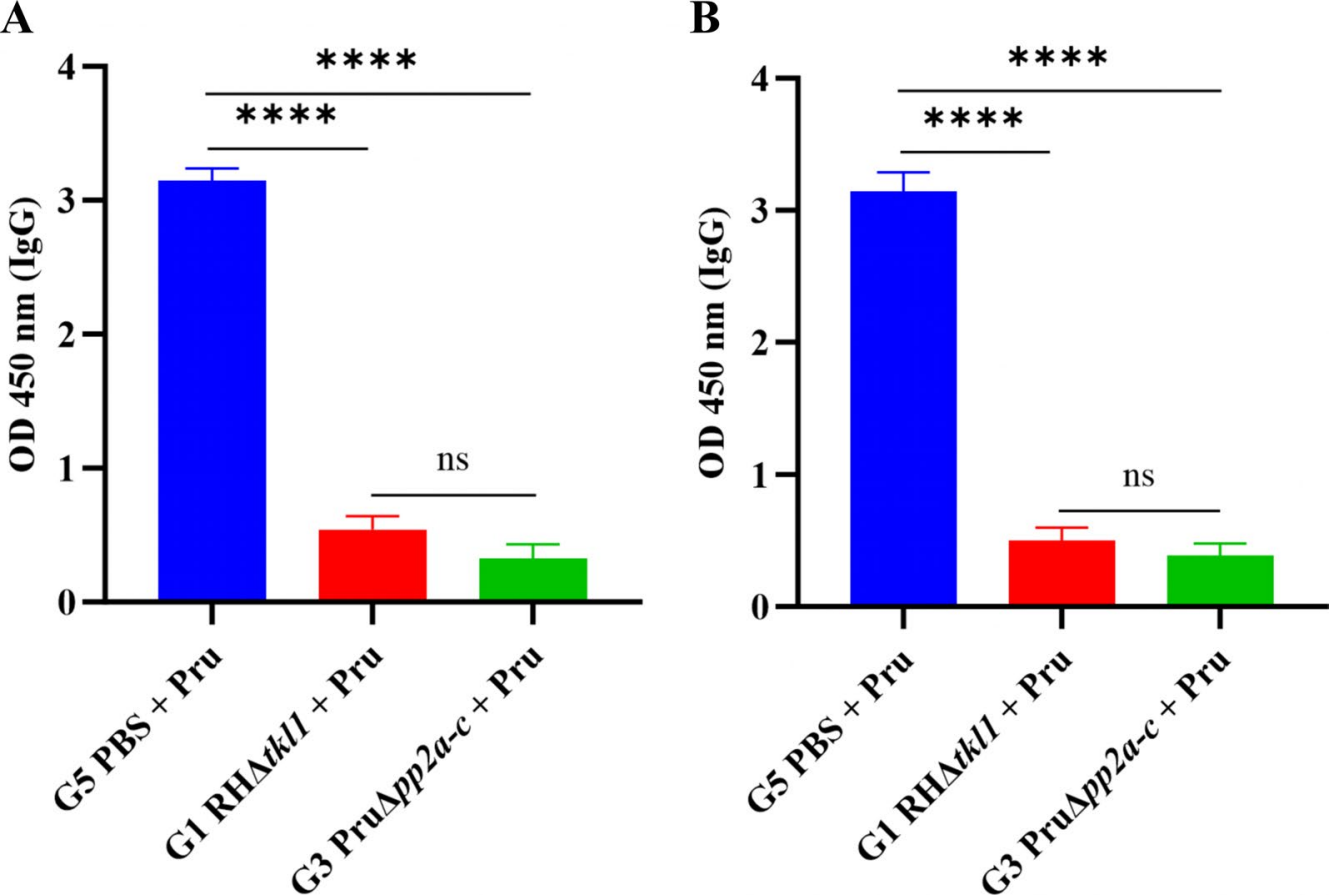
*Toxoplasma gondii*-specific IgG antibodies in mice inoculated with sheep tissues. **A** Serum IgG levels in mice inoculated with brain tissue from sheep in groups G1 (vaccinated with RHΔ*tkl1* tachyzoites, challenged), G3 (vaccinated with PruΔ*pp2a-c* tachyzoites, challenged), and G5 (non-vaccinated, challenged). **B** Serum IgG levels in mice inoculated with visceral tissue from the sheep in groups G1, G3, and G5. ****P < 0.0001, ***P < 0.001, **P < 0.01, *P < 0.05

Compared to mice inoculated with brain tissue from G5 sheep, mice inoculated with brain tissue from G1 and G3 sheep showed significantly lower levels of *T. gondii*-specific IgG antibody (*P* < 0.0001; Fig. 7A). Similarly, a significant decrease in *T. gondii* IgG antibody level was also observed in mice inoculated with visceral tissues from G1 and G3 sheep, compared to those inoculated with visceral tissues from the G5 group (*P* < 0.0001) (Fig. 7B).

## Discussion

Toxoplasmosis is a globally prevalent zoonotic disease that not only poses a serious threat to human health but also causes significant harm to the development of the livestock industry, particularly affecting economically important animals such as sheep and pigs [56]. In sheep, infection with *T. gondii* can lead to acute symptoms such as fever, decreased appetite, and neurological disorders (e.g., circling and convulsions), or form cysts within their bodies, rendering infected animals potential reservoirs of transmission [27]. In pregnant ewes, infection may cause abortion, stillbirth, and recurrent miscarriages, severely impairing reproductive performance [27]. The pathogen can also be transmitted vertically through the placenta, leading to congenital infections in fetuses [27]. Additionally, meat from infected sheep may contain cysts, and consumption of undercooked mutton therefore represents a risk for human infection [27]. Therefore, effective prevention and control of toxoplasmosis remain critical issues in public health and veterinary medicine. Attenuated live vaccines developed through gene knockout represent a promising strategy against toxoplasmosis [35].

Following immunization with either RHΔ*tkl1* or PruΔ*pp2a-c* tachyzoites, the sheep exhibited only a transient rise in body temperature without other clinical signs (Fig. 2A). This suggests that the *T. gondii* gene knockout attenuated strains RHΔ*tkl1* and PruΔ*pp2a-c* are relatively safe for inoculated sheep and do not cause serious pathological damage. Following challenge with *T. gondii* Pru strain oocysts, the body temperature of sheep in group G5 remained elevated for approximately 7 days, while in groups G1 and G3, the elevated body temperature persisted for about 3 days (Fig. 2B). A similar febrile response—with peak temperatures nearing 42 °C—has been reported in sheep orally challenged with 5 × 10^5^ oocysts of the *T. gondii* M4 strain following immunization with the S48 strain [36]. Following the boosted challenge with *T. gondii* Pru strain tachyzoites, the temperature profiles in groups G1, G3, and G5 were consistent with those observed after the primary oocyst challenge (Fig. 2C). Notably, challenge at 28 dpv triggered a stronger and more sustained increase in anti-*T. gondii* IgG antibodies, reflecting the activation of an antigen-specific memory response and a robust secondary immune reaction.

Histopathological examination revealed extensive inflammation changes, including lymphocytic infiltration and cellular necrosis in the organs (heart, liver, lung) of sheep in the G5 group (Additional file 2: Fig. S2; Additional file 3: Fig. S3). In contrast, lesions severity was significantly reduced in the groups G1 and G3, where only localized tissue edema or minimal inflammatory cell infiltration were observed (Additional file 2: Fig. S2; Additional file 3: Fig. S3). These findings indicate that these gene knockout strains effectively attenuated the pathogenicity of the parasite, consistent with previous reports that that deletion of the TKL1 gene significantly reduces the strain pathogenicity to the host [41]. Notably, increased lymphocytic infiltration—particularly in the myocardial layer and perivascular regions of the lung—was evident in tissues from groups G1 and G3 (Additional file 2: Fig. S2; Additional file 3: Fig. S3), indicating activation of cellular immunity. A marked increase in lymphocytic infiltration (particularly in the myocardial layer and perivascular areas of the lung) was noted in the tissues of sheep from the G1 and G3 groups (Additional file 2: Fig. S2; Additional file 3: Fig. S3), suggesting the activation of cellular immunity. This response is likely mediated by T-cells, which can suppress parasite proliferation through cytokine secretion such as IFN-γ [57]. Immunohistochemical analysis revealed that group G5 showed significantly higher values than groups G1 and G3 across all five parameters (positive cell ratio, positive cell density, H-Score, mean optical density, and immunoreactive score) assessed (Fig. 4). Together, these results demonstrate that vaccination of the attenuated strains RHΔ*tkl1* and PruΔ*pp2a-c* substantially reduced parasite antigen load in brain tissue.

Microscopic examination of brain cysts in sheep showed a significant reduction in the cyst numbers in the groups G1 and G3 compared to group G5 (*P* < 0.01) (Fig. 5B). Notably, the cyst count in group G3 was significantly lower than that in group G1 (*P* < 0.01) (Fig. 5B). This may be associated with the critical role of the PP2A-C gene in the bradyzoite conversion of *T. gondii*, as deletion of any subunit within the parasite's protein phosphatase 2A holoenzyme can prevent cyst formation [42]. However, the fact that *T. gondii* cysts still formed in the brain tissue of G1 and G5, which received only the gene knockout attenuated strains, suggests that neither of these two attenuated strains is completely safe.

In our study, sheep were challenged with *T. gondii* Pru strain oocysts on day 28, and then re-challenged with *T. gondii* Pru strain tachyzoites on day 70. The secondary challenge was performed because we aimed to evaluate whether two gene knockout attenuated strains of *T. gondii* confer protective effects against different developmental stages of *T. gondii* in sheep. However, this also constitutes a limitation of the experimental design, as it does not allow us to better assess the protective efficacy of the two gene knockout attenuated strains against each individual developmental stage of *T. gondii* infection in sheep. After sheep were immunized with RHΔ*tkl1*, the peak level of *T. gondii*-specific IgG antibody was significantly higher than that observed after immunization with PruΔ*pp2a-c*. However, following the secondary challenge with *T. gondii* Pru tachyzoites on day 70, the *T. gondii*-specific antibody levels in the PruΔ*pp2a-c*-immunized group remained elevated for a longer duration and continued to be higher than those in the RHΔ*tkl1*-immunized group until the end of the experiment. Additionally, the number of brain cysts in sheep in group G3 was significantly lower than that in group G1, indicating that PruΔ*pp2a‑c* was more effective at inhibiting the formation of brain cysts in sheep than RHΔ*tkl1*. These results suggest that the immune effect induced by PruΔ*pp2a-c* is superior to that of RHΔ*tkl1*.

In summary, this study systematically evaluated, for the first time in a sheep model, the safety and immuno-protective efficacy of the gene knockout attenuated strains RHΔ*tkl1* and PruΔ*pp2a-c*. The results demonstrate that both gene knockout attenuated strains have a certain degree of safety and are promising candidate vaccines against *T. gondii*. Notably, PruΔ*pp2a-c* exhibited superior potential in controlling chronic infection. Its ability to reduce cyst formation may lower parasite contamination in meat products, thereby decreasing the risk of foodborne transmission to humans. However, it should be noted that a small number of cysts were still detected in the tissues of immunized sheep, indicating that while these gene knockout attenuated strains can mitigate infection severity, they cannot completely prevent cyst formation. Future research could explore strategies such as multi-gene knockout or combination with adjuvants to enhance their protective efficacy. This study provides a solid foundation for the clinical translation of a toxoplasmosis vaccine for sheep and offers promising candidate strains along with relevant experimental data to support the development of livestock vaccines against toxoplasmosis.

## Conclusions

In conclusion, this study demonstrates that the attenuated* T. gondii* strains, RHΔ*tkl1* and PruΔ*pp2a-c*, confer effective immuno-protection against toxoplasmosis in sheep. Immunization with tachyzoites of either attenuated strain significantly alleviated acute clinical signs, reduced tissue lesions, and decreased parasite burden following challenge infections. The vaccinated animals developed high and sustained levels of *T. gondii*-specific IgG antibodies, which persisted throughout the 112-day experiment. Notably, PruΔ*pp2a-c* exhibited a superior efficacy in suppressing cyst formation. Together, these findings indicate that both attenuated strains can elicit robust humoral and cellular immune responses in sheep and provide durable protective immunity against *T. gondii* infection. Therefore, both RHΔ*tkl1* and PruΔ*pp2a-c* represent promising candidate strains for a live-attenuated vaccine against sheep toxoplasmosis.

## Supplementary Information

Additional file 1.

Additional file 2.

Additional file 3.

Additional file 4.

Additional file 5.

## Data Availability

The datasets supporting the findings of this article are included within the paper and its supplementary materials.

## Abbreviations

DPV: Days post-vaccination
HFF: Human foreskin fibroblast
FBS: Fetal bovine serum
HE: Hematoxylin and eosin
IHC: Staining and immunohistochemistry
ELISA: Enzyme-linked immunosorbent assay
OD: Optical density
NBF: Neutral buffered formalin
IRS: Immunoreactive score
RT-qPCR: Reverse transcription quantitative polymerase chain reaction
QPCR: Quantitative polymerase chain reaction
CT: Cycle threshold
PBST: Phosphate buffered saline with tween
BSA: Bovine serum albumin
TMB: 3,3′,5,5′-Tetramethylbenzidine
IgG: Immunoglobulin G

## References

[1] Kochanowsky JA, Koshy AA. Toxoplasma gondii. Curr Biol. 2018;28:R770–1. 30040931 10.1016/j.cub.2018.05.035

[2] Kooij TW, Janse CJ, Waters AP. Plasmodium post-genomics: better the bug you know? Nat Rev Microbiol. 2006;4:344–57. 16582929 10.1038/nrmicro1392

[3] Zhang Y, Jiang P, Yin J, Zhu G. Pathogenicity and virulence of *Cryptosporidium*. Virulence. 2025;16:2597642. 41319271 10.1080/21505594.2025.2597642 PMC12688253

[4] Elsheikha HM, Marra CM, Zhu XQ. Epidemiology, pathophysiology, diagnosis, and management of cerebral toxoplasmosis. Clin Microbiol Rev. 2021;34:e00115-00119. 33239310 10.1128/CMR.00115-19 PMC7690944

[5] Dubey JP, Lindsay DS, Speer CA. Structures of *Toxoplasma gondii* tachyzoites, bradyzoites, and sporozoites and biology and development of tissue cysts. Clin Microbiol Rev. 1998;11:267–99. 9564564 10.1128/cmr.11.2.267 PMC106833

[6] Mendez OA, Koshy AA. *Toxoplasma gondii*: Entry, association, and physiological influence on the central nervous system. PLoS Pathog. 2017;13:e1006351. 28727854 10.1371/journal.ppat.1006351 PMC5519211

[7] Attias M, Teixeira DE, Benchimol M, Vommaro RC, Crepaldi PH, Souza WD. The life-cycle of *Toxoplasma gondii* reviewed using animations. Parasit Vectors. 2020;13:588. 33228743 10.1186/s13071-020-04445-z PMC7686686

[8] Hill DE, Chirukandoth S, Dubey JP. Biology and epidemiology of *Toxoplasma gondii* in man and animals. Anim Health Res Rev. 2005;6:41–61. 16164008 10.1079/ahr2005100

[9] Hill D, Dubey JP. *Toxoplasma gondii*: transmission, diagnosis and prevention. Clin Microbiol Infect. 2002;8:634–40. 12390281 10.1046/j.1469-0691.2002.00485.x

[10] Hopper AT, Brockman A, Wise A, Gould J, Barks J, Radke JB, et al. Discovery of selective *Toxoplasma gondii* dihydrofolate reductase inhibitors for the treatment of toxoplasmosis. J Med Chem. 2019;62:1562–76. 30624926 10.1021/acs.jmedchem.8b01754 PMC6571122

[11] Dunn D, Wallon M, Peyron F, Petersen E, Peckham C, Gilbert R. Mother-to-child transmission of toxoplasmosis: risk estimates for clinical counselling. Lancet. 1999;353:1829–33. 10359407 10.1016/S0140-6736(98)08220-8

[12] Robert-Gangneux F, Dardé ML. Epidemiology of and diagnostic strategies for toxoplasmosis. Clin Microbiol Re. 2012;25:264–96. 10.1128/CMR.05013-11 PMC3346298 22491772

[13] Jones JL, Muccioli C, Belfort RJ, Holland GN, Roberts JM, Silveira C. Recently acquired *Toxoplasma gondii* infection. Brazil Emerg Infect Dis. 2006;12:582–7. 16704805 10.3201/eid1204.051081 PMC3294697

[14] Su C, Khan A, Zhou P, Majumdar D, Ajzenberg D, Dardé ML, et al. Globally diverse *Toxoplasma gondii* isolates comprise six major clades originating from a small number of distinct ancestral lineages. Proc Natl Acad Sci U S A. 2012;109:5844–9. 22431627 10.1073/pnas.1203190109 PMC3326454

[15] Khan A, Dubey JP, Su C, Ajioka JW, Rosenthal BM, Sibley LD. Genetic analyses of atypical *Toxoplasma gondii* strains reveal a fourth clonal lineage in North America. Int J Parasitol. 2011;41:645–55. 21320505 10.1016/j.ijpara.2011.01.005 PMC3081397

[16] Lv QB, Ma H, Wei J, Qin YF, Qiu HY, Ni HB, et al. Changes of gut microbiota structure in rats infected with *Toxoplasma gondii*. Front Cell Infect Microbiol. 2022;12:969832. 35967867 10.3389/fcimb.2022.969832 PMC9366923

[17] Velmurugan GV, Dubey JP, Su C. Genotyping studies of *Toxoplasma gondii* isolates from Africa revealed that the archetypal clonal lineages predominate as in North America and Europe. Vet Parasitol. 2008;155:314–8. 18583059 10.1016/j.vetpar.2008.04.021

[18] Dubey JP, Cerqueira-Cézar CK, Murata FHA, Kwok OCH, Yang YR, Su C. All about toxoplasmosis in cats: the last decade. Vet Parasitol. 2020;283:109145. 32645556 10.1016/j.vetpar.2020.109145

[19] Wang L, He LY, Meng DD, Chen ZW, Wen H, Fang GS, et al. Seroprevalence and genetic characterization of *Toxoplasma gondii* in cancer patients in Anhui Province. Eastern China Parasit Vectors. 2015;8:162. 25889184 10.1186/s13071-015-0778-5 PMC4379604

[20] Jiang HH, Huang SY, Zhou DH, Zhang XX, Su C, Deng SZ, et al. Genetic characterization of *Toxoplasma gondii* from pigs from different localities in China by PCR-RFLP. Parasit Vectors. 2013;6:227. 23919620 10.1186/1756-3305-6-227 PMC3750303

[21] Li M, Mo XW, Wang L, Chen H, Luo QL, Wen HQ, et al. Phylogeny and virulence divergency analyses of *Toxoplasma gondii* isolates from China. Parasit Vectors. 2014;7:133. 24678633 10.1186/1756-3305-7-133 PMC3986613

[22] Matta SK, Rinkenberger N, Dunay IR, Sibley LD. *Toxoplasma gondii* infection and its implications within the central nervous system. Nat Rev Microbiol. 2021;19:467–80. 33627834 10.1038/s41579-021-00518-7

[23] Pappas G, Roussos N, Falagas ME. Toxoplasmosis snapshots: global status of *Toxoplasma gondii* seroprevalence and implications for pregnancy and congenital toxoplasmosis. Int J Parasitol. 2009;39:1385–94. 19433092 10.1016/j.ijpara.2009.04.003

[24] Dong H, Su R, Lu Y, Wang M, Liu J, Jian F, et al. Prevalence, risk factors, and genotypes of *Toxoplasma gondii* in food animals and humans (2000–2017) from China. Front Microbiol. 2018;9:2108. 30254613 10.3389/fmicb.2018.02108 PMC6141634

[25] Tenter AM, Heckeroth AR, Weiss LM. *Toxoplasma gondii*: from animals to humans. Int J Parasitol. 2000;30:1217–58. 11113252 10.1016/s0020-7519(00)00124-7 PMC3109627

[26] Dubey JP. Toxoplasmosis in pigs–the last 20 years. Vet Parasitol. 2009;164:89–103. 19559531 10.1016/j.vetpar.2009.05.018

[27] Dubey JP. Toxoplasmosis in sheep–the last 20 years. Vet Parasitol. 2009;163:1–14. 19395175 10.1016/j.vetpar.2009.02.026

[28] Jones JL, Dubey JP. Foodborne toxoplasmosis. Clin Infect Dis. 2012;55:845–51. 22618566 10.1093/cid/cis508

[29] Dubey JP. The history of Toxoplasma gondii–the first 100 years. J Eukaryot Microbiol. 2008;55:467–75. 19120791 10.1111/j.1550-7408.2008.00345.x

[30] Duncanson P, Terry RS, Smith JE, Hide G. High levels of congenital transmission of *Toxoplasma gondii* in a commercial sheep flock. Int J Parasitol. 2001;31:1699–703. 11730799 10.1016/s0020-7519(01)00282-x

[31] Castaño P, Fuertes M, Regidor-Cerrillo J, Ferre I, Fernández M, Ferreras MC, et al. Experimental ovine toxoplasmosis: influence of the gestational stage on the clinical course, lesion development and parasite distribution. Vet Res. 2016;47:43. 26983883 10.1186/s13567-016-0327-z PMC4793618

[32] Buxton D. Protozoan infections (*Toxoplasma gondii*, *Neospora caninum* and *Sarcocystis* spp.) in sheep and goats: recent advances. Vet Res. 1998;29:289–310. 9689743

[33] Dubey JP, Hill DE, Jones JL, Hightower AW, Kirkland E, Roberts JM, et al. Prevalence of viable *Toxoplasma gondii* in beef, chicken, and pork from retail meat stores in the United States: risk assessment to consumers. J Parasitol. 2005;91:1082–93. 16419752 10.1645/GE-683.1

[34] Ahaduzzaman M, Hasan T. Seroprevalence of *Toxoplasma gondii* infection in sheep and goats from different geographical regions of the world: systematic review and meta-analysis. Transbound Emerg Dis. 2022;69:3790–822. 36345796 10.1111/tbed.14753

[35] Wang JL, Zhang NZ, Li TT, He JJ, Elsheikha HM, Zhu XQ. Advances in the development of anti-*Toxoplasma gondii* vaccines: challenges, opportunities, and perspectives. Trends Parasitol. 2019;35:239–53. 30718083 10.1016/j.pt.2019.01.005

[36] Katzer F, Canton G, Burrells A, Palarea-Albaladejo J, Horton B, Bartley PM, et al. Immunization of lambs with the S48 strain of *Toxoplasma gondii* reduces tissue cyst burden following oral challenge with a complete strain of the parasite. Vet Parasitol. 2014;205:46–56. 25062897 10.1016/j.vetpar.2014.07.003

[37] Zhang NZ, Chen J, Wang M, Petersen E, Zhu XQ. Vaccines against *Toxoplasma gondii*: new developments and perspectives. Expert Rev Vaccines. 2013;12:1287–99. 24093877 10.1586/14760584.2013.844652

[38] Zhang Y, Li D, Lu S, Zheng B. Toxoplasmosis vaccines: what we have and where to go? NPJ vaccines. 2022;7:131. 36310233 10.1038/s41541-022-00563-0 PMC9618413

[39] Mévélec MN, Ducournau C, Bassuny Ismael A, Olivier M, Sèche E, Lebrun, et al. Mic1-3 knockout *Toxoplasma gondii* is a good candidate for a vaccine against *T. gondii*-induced abortion in sheep. Vet Res. 2010;41:Mic1. 10.1051/vetres/2010021 PMC2865876 20385082

[40] Buxton D, Innes EA. A commercial vaccine for ovine toxoplasmosis. Parasitology. 1995;110:S11–6. 7784124 10.1017/s003118200000144x

[41] Wang JL, Liang QL, Li TT, He JJ, Bai MJ, Cao XZ, et al. *Toxoplasma gondii* tkl1 deletion mutant is a promising vaccine against acute, chronic, and congenital toxoplasmosis in mice. J Immunol. 2020;204:1562–70. 31996457 10.4049/jimmunol.1900410

[42] Wang JL, Li TT, Elsheikha HM, Liang QL, Zhang ZW, Wang M, et al. The protein phosphatase 2A holoenzyme is a key regulator of starch metabolism and bradyzoite differentiation in *Toxoplasma gondii*. Nat Commun. 2022;13:7560. 36476594 10.1038/s41467-022-35267-5 PMC9729606

[43] Xie SC, Lv YH, Wang M, Zheng XN, Wang JL, Fu BQ, et al. Live-attenuated *Toxoplasma gondii* PruΔ*pp2a-c* mutant elicits protective immunity against toxoplasmosis in mice and cats. Int J Parasitol. 2026;56:104714. 40783178 10.1016/j.ijpara.2025.08.002

[44] Burrells A, Benavides J, Cantón G, Garcia JL, Bartley PM, Nath M, et al. Vaccination of pigs with the S48 strain of Toxoplasma gondii–safer meat for human consumption. Vet Res. 2015;46:47. 25928856 10.1186/s13567-015-0177-0 PMC4415212

[45] Wu Q, Zhang Z, Chu H, Xia B, Li W, Ding J, et al. A novel combined quadrivalent self-amplifying mRNA-LNP vaccine provokes protective immunity against acute and chronic toxoplasmosis in mice. Infect Dis Poverty. 2025;14:55. 40551280 10.1186/s40249-025-01332-6 PMC12183821

[46] Buxton D, Miller HR, Finlayson J, Wallace GR. *Toxoplasma gondii*: its effect on the ovine popliteal lymph node. J Med Microbiol. 1981;14:435–42. 7310845 10.1099/00222615-14-4-435

[47] Benavides J, Maley S, Pang Y, Palarea J, Eaton S, Katzer F, et al. Development of lesions and tissue distribution of parasite in lambs orally infected with sporulated oocysts of *Toxoplasma gondii*. Vet Parasitol. 2011;179:209–15. 21440372 10.1016/j.vetpar.2011.03.001

[48] Benonisson H, Altıntaş I, Sluijter M, Verploegen S, Labrijn AF, Schuurhuis DH, et al. CD3-Bispecific antibody therapy turns solid tumors into inflammatory sites but does not install protective memory. Mol Cancer Ther. 2019;18:312–22. 30381448 10.1158/1535-7163.MCT-18-0679

[49] Yin JZ, Shi XQ, Wang MD, Du H, Zhao XW, Li B, et al. Arsenic trioxide elicits anti-tumor activity by inhibiting polarization of M2-like tumor-associated macrophages via Notch signaling pathway in lung adenocarcinoma. Int Immunopharmacol. 2023;117:109899. 36827926 10.1016/j.intimp.2023.109899

[50] Zhao R, Zhou Y, Shi H, Ye W, Lyu Y, Wen Z, et al. Effect of gestational diabetes on postpartum depression-like behavior in rats and its mechanism. Nutrients. 2022;14:1229. 35334886 10.3390/nu14061229 PMC8953401

[51] Wang JL, Bai MJ, Elsheikha HM, Liang QL, Li TT, Cao XZ, et al. Novel roles of dense granule protein 12 (GRA12) in *Toxoplasma gondii* infection. FASEB J. 2020;3:3165–78. 10.1096/fj.201901416RR 31908049

[52] Montazeri M, Ebrahimzadeh MA, Ahmadpour E, Sharif M, Sarvi S, Daryani A. Evaluation of propranolol effect on experimental acute and chronic toxoplasmosis using quantitative PCR. Antimicrob Agents Chemother. 2016;60:7128–33. 27645234 10.1128/AAC.01323-16 PMC5118991

[53] Dubey JP. Refinement of pepsin digestion method for isolation of *Toxoplasma gondii* from infected tissues. Vet Parasitol. 1998;74:75–7. 9493311 10.1016/s0304-4017(97)00135-0

[54] Yang J, Yang C, Qian J, Li F, Zhao J, Fang R. *Toxoplasma gondii* α-amylase deletion mutant is a promising vaccine against acute and chronic toxoplasmosis. Microb Biotechnol. 2020;13:2057–69. 32959958 10.1111/1751-7915.13668 PMC7533317

[55] Li J, Kang Y, Wu ZX, Yang SF, Tian YY, Zhu XQ, et al. Live-attenuated PruΔ*gra72* strain of *Toxoplasma gondii* induces strong protective immunity against acute and chronic toxoplasmosis in mice. Parasit Vectors. 2024;17:377. 39237959 10.1186/s13071-024-06461-9 PMC11378421

[56] Zhao XY, Ewald SE. The molecular biology and immune control of chronic *Toxoplasma gondii* infection. J Clin Invest. 2020;130:3370–80. 32609097 10.1172/JCI136226 PMC7324197

[57] Bartley PM, Burrells A, Benavides J, Canton G, Garcia JL, Thomson J, et al. Cell mediated and innate immune responses in pigs following vaccination and challenge with *Toxoplasma* parasites. Vet Parasitol. 2019;275:108963. 31669836 10.1016/j.vetpar.2019.108963

